# Collagen characteristics affect the texture of pork *Longissimus* and *Biceps femoris*

**DOI:** 10.1093/tas/txac129

**Published:** 2022-09-20

**Authors:** Xiying Li, Minh Ha, Robyn D Warner, Frank R Dunshea

**Affiliations:** Faculty of Veterinary and Agricultural Sciences, The University of Melbourne, Parkville, VIC 3010, Australia; Faculty of Veterinary and Agricultural Sciences, The University of Melbourne, Parkville, VIC 3010, Australia; Faculty of Veterinary and Agricultural Sciences, The University of Melbourne, Parkville, VIC 3010, Australia; Faculty of Veterinary and Agricultural Sciences, The University of Melbourne, Parkville, VIC 3010, Australia; Faculty of Biological Sciences, University of Leeds, Leeds LS2 9JT, UK

**Keywords:** meat texture, collagen, intramuscular fat, correlation, muscles

## Abstract

Connective tissue is considered to influence the toughness of pork, but most studies on connective tissue focus on the loin muscle. Cooking loss, Warner–Bratzler shear force (WBSF), texture profile analysis (hardness, springiness, chewiness, resilience, and cohesiveness), total and insoluble collagen content, the percentage of type III collagen relative to the total of type I and III collagen, proteoglycan content and intramuscular fat (IMF) content were measured for pork *Biceps femoris* (BF) and *Longissimus thoracis et lumborum* (LTL) and Pearson’s correlation was performed. The results showed that BF had higher WBSF, resilience, total, and insoluble collagen content than LTL (*P* < 0.05). When considering both muscles, total and insoluble collagen content were positively correlated (*P* < 0.05) with WBSF (*r* = 0.47 and 0.49, respectively), hardness (*r* = 0.49 and 0.50, respectively), cohesiveness (*r* = 0.50 and 0.50, respectively), chewiness (*r* = 0.58 and 0.59, respectively) and resilience (*r* = 0.63 and 0.63, respectively). The percentage of type III collagen content was negatively correlated (*P* < 0.05) with WBSF (*r* = −0.68) and hardness (*r* = −0.58). Collagen content was correlated (*P* < 0.05) with WBSF (*r* = 0.75) and hardness (*r* = 0.61) in LTL and with cohesiveness (*r* = 0.74) and resilience (*r* = 0.63) in BF. Collagen characteristics differ between muscles and contribute to pork texture in both LTL and BF.

## Teaser

Total and insoluble collagen content as well as the percentage of type III collagen contributed to the toughness of both pork *Longissimus thoracis et lumborum*and *Biceps femoris*.

## INTRODUCTION

Meat texture is an important attribute for consumers to assess meat quality which can be affected by muscle composition, such as connective tissue and intramuscular fat (IMF). Connective tissue has been considered to define the “background toughness” of meat (Sentandreu et al., 2002) and different muscles vary in connective tissue characteristics and meat texture. For example, pork muscles from the leg primal were reported to be tougher than the loin, with a higher collagen content (Nishimura et al., 2009; Petersen et al., 1997). However, Wheeler et al. (2000) found that the correlation between collagen content and pork sensory tenderness was only significant when all muscles (*Longissimus lumborum, Biceps femoris, Semimembranosus, Semitendinosus,* and *Triceps brachii*) were taken into consideration and the correlation coefficients varied between muscles. A recent meta-analysis showed that collagen content was only correlated with sensory tenderness in muscles other than loin in beef (Li et al., 2022). There were insufficient published data to conduct a similar meta-analysis on pork.

Most studies on the relationship between collagen content and pork tenderness were conducted on loin muscles. Only a few studies have investigated the collagen characteristics, such as type and solubility, of different pork muscles and their relationship with instrumental meat texture. In addition, not only collagen content and solubility but also collagen isoforms and proteoglycan content can influence meat tenderness. IMF has been considered to influence pork tenderness, but the effects varied in previous studies (Fortin et al., 2005; Rincker et al., 2008). Therefore, this study aimed to 1) determine the texture, collagen characteristics and IMF content of pork from the *Longissimus thoracis et lumborum* (LTL) and *Biceps femoris* (BF), and 2) to find out how collagen and IMF contribute to the texture of the two muscles. The loin and leg cuts are commonly consumed by Australian consumers and the BF is the biggest muscle in the leg cut. Investigation of the properties of these two muscles provides preliminary information for the pork industry. We hypothesized that 1) pork BF has a higher collagen content than LTL; and 2) that collagen characteristics and IMF content are correlated with pork texture, with higher correlation coefficients in BF compared to loin.

## MATERIALS AND METHODS

### Sample Preparation

All slaughterhouses followed the Australian Standard AS 4696:2007 Hygienic Production and Transportation of Meat and Meat Products for Human Consumption, Industry Animal Welfare Standards for Livestock Processing Establishments Preparing Meat for Human Consumption—Third Edition and Model Code of Practice for the Welfare of Animals: Livestock at Slaughtering Establishments. Pork loin (*n* = 11) and leg cuts (*n* = 12) were purchased from a local butcher, which was not the slaughter site, at four different times. All the cuts were from different animals. The same cuts were from the same locations on the carcass. The *Biceps femoris* (BF) and *Longissimus thoracis et lumborum* (LTL) were excised from the leg and loin cuts, respectively. From each muscle, a 2 cm thick piece was cut out from the middle line of the muscle towards the posterior end. From this piece, 90 g of muscle was randomly collected for freeze-drying and 3 g of finely chopped muscle (in triplicate) was randomly collected for collagen extraction. From the middle line of each muscle towards the anterior end, a cuboid of 55 × 55 × 38 mm weighing approximately 120 g was cut and vacuum packed for texture analysis. All samples were kept at −20°C until further analysis.

### Cooking Loss, Warner–Bratzler Shear Force, and Texture Profile Analysis

Vacuum packed pork was cooked from frozen without thawing in an 80 °C water bath. The internal temperature was monitored using a reference sample from LTL with the same dimension and weight. The reference sample was inserted with a thermocouple probe before being frozen. It was placed in a vacuum pack pouch and cooked with other samples. When the internal temperature of the reference sample reached 71 °C, all samples were taken out from the water bath and then cooled in iced water for 30 min. Cooked samples were stored at 2 °C overnight before texture measurement. On the next day, the cooked pork was removed from the packaging and excessive fluid was absorbed using a paper towel. Cooking loss was calculated as follow:


$$\begin{array}{l} Cooking\;\;\;loss\left(\%\right)=\left(W_{b}-W_{c}\right)/W_{b}\times 100\% \end{array}$$

where *W*_b_ is the sample weight before cooking (g) and *W*_c_ is the sample weight after cooking (g).

Warner–Bratzler shear force (WBSF) and texture profile analysis (TPA) were measured using the Lloyd Texture Analyser (AMETEK, Berwyn, Pennsylvania USA) with a 500 N load cell. The cooked sample was cut along the muscle fibers to obtain steaks 1 cm thick. One steak was kept for TPA measurement. For WBSF measurement, the steaks were then cut along the muscle fibers to obtain cuboids of 1 cm^2^ × 3 cm. The cuboids were cut with a V-shape cutting blade at an extension rate of 300 mm/min and 6 cuboids were measured for each sample. WBSF was expressed in units of Newton (N). For texture profile analysis, a 6 mm cylindrical probe was used to compress the 1 cm thick steaks. Texture profile was obtained with a double bite process. The test speed was 50 mm/min and the wait time was 0.1 s prior to the start of the second compression. The sample was compressed to 80% of its height. The compression force was perpendicular to the muscle fibers. Five locations in each steak were measured and visible fat was carefully avoided. Hardness (N), adhesiveness (N mm), cohesiveness, springiness, resilience, and chewiness (N) were obtained from the NEXYGENPlus program (AMETEK, Berwyn, Pennsylvania USA).

### Total and Insoluble Collagen Content

The determination of total and soluble collagen followed the AOAC method 990.26 (Kolar, 1990) described by Starkey et al. (2015). Triplicate 0.2 g freeze-dried meat powder was used for analysis. A conversion factor of 7.25 was used to calculate collagen content from hydroxyproline content. Insoluble collagen content was calculated by subtracting soluble collagen content from total collagen content. Total and insoluble collagen content was expressed as mg/g of fresh meat.

### Collagen Types I and III Ratio

The extraction of collagen followed the method described by Muralidharan et al. (2013) with some modifications. The extraction process was conducted at 4 °C. Three grams of finely chopped meat sample (triplicate) was washed with 0.8 M NaCl (1:6 w/v) for 10 min three times. The sample was then washed in 0.1 M NaOH (1:10 w/v) with shaking for 48 h with changes of solution every 16 h, followed by washing with water. Then, the sample was mixed with 0.5 M acetic acid (1:15 w/v) containing 0.1% (w/v) pepsin and was shaken for 24 h. The mixture was filtered through glass wool and the supernatant was salted out by adding NaCl to a final concentration of 2.6 M and precipitates were collected by centrifuging at 4,752 *g* for 30 min. The precipitate was dissolved in 0.5 M acetic acid and dialyzed against 0.02M Na_2_HPO_4_ for 24 h with the change of solution three times. Dialyzed samples were freeze-dried and stored at −20 °C before further analysis.

Quantification of types I and III collagen was through SDS–PAGE. SDS–PAGE was performed with a 7.5% separating gel and a 4% stacking gel as described by Cho et al. (2017). The percentage of type III collagen was calculated using a densitometry method according to Burson and Hunt (1986a). The peak areas were adjusted for the number of alpha chains (α1) in types I and III collagen. The percentage of type III collagen was calculated as follows:


$$\begin{array}{ll} \; & Percentage\;of\;type\;III\;collagen=\\ & \frac{Peak\;area\;of\;\alpha 1\left(III\right)}{Peak\;area\;of\;\alpha 1\left(I\right)x1.5\;+Peak\;area\;of\;\alpha 1\left(III\right)}\times 100\% \end{array}$$

### Proteoglycan Content

The proteoglycan content assay followed that described by Wang et al. (2016) and Coulson-Thomas and Ferreira (2014) with some modifications. Dimethylmethylene blue (DMMB) reagent was prepared by dissolving 16 mg DMMB in 95 mL of 0.1 M acetic acid with 3.04 g glycine and 1.6 g NaCl, and the volume was made up to 1 L water (final pH 3.0). Freeze-dried sample (200 mg) was digested with papain (≥3 U/mg, 1 mg/mL) in 5 mL of phosphate buffer (50 mM, pH 6.5) containing 2 mM N-acetyl cysteine and 2 mM EDTA at 65 °C for 16 h. Then the solution was centrifuged at 4,752 *g* for 15 min at 4 °C, filtered, and 1 mL of supernatant was diluted with 1 mL of phosphate buffer. Chondroitin 4-sulfate was used as standard with a concentration of 0, 5, 10, 15, 20, and 25 µg/mL. In a microplate, 20 µL of sample and standard was loaded and 200 µL of DMMB solution was added to the wells. The microplate was shaken for 5 s and the absorbance at 525 nm was immediately measured. Proteoglycan content was expressed as mg/g collagen.

### Intramuscular Fat Content

Intramuscular fat (IMF) content was determined by the AOAC method 983.18 (AOAC, 1995) with some modifications. Briefly, triplicate 3.5 g freeze-dried samples were powdered and wrapped in a folded Whatman no. 1 filter paper before conducting Soxhlet extraction. The extraction solvent was diethyl ether. Intramuscular fat content was expressed as percent fat per g of fresh meat.

### Statistical Analysis

Unbalanced analysis of variance (ANOVA) was conducted on cooking loss, texture, collagen characteristics, and IMF content with purchase day as a blocking factor using GenStat (16th Edition, VSN International). Correlation matrices were obtained using RStudio (RStudio, PBC) between cooking loss, texture parameters, IMF, and collagen characteristics of both and individual muscles. Principal component analysis (PCA) was performed on both muscles in RStudio (RStudio, PBC) and the PCA loading plot was plotted.

## RESULTS

As shown in Table 1, the pork BF showed higher WBSF (31.38 vs. 26.35 N, *P* = 0.004) and resilience (0.453 vs. 0.431, *P* = 0.047) compared to the LTL. Springiness of BF tended to be slightly higher than LTL (0.765 vs. 0.737, *P* = 0.094). However, the muscles did not differ in cooking loss, hardness, cohesiveness, adhesiveness, or chewiness. The BF had higher total collagen (6.67 vs, 4.95 mg/g, *P* < 0.001) and insoluble collagen (6.15 vs. 4.52 mg/g, *P* < 0.001) contents than the LTL (Table 1). No significant difference was found in the percentage of type III collagen, proteoglycan content, or IMF content between the two muscles.

**Table 1.** Cooking loss, Warner–Bratzler shear force (WBSF), texture profile analysis, and chemical components of pork *Longissimus thoracis et lumborum* (LTL, *N* = 11) and *Biceps femoris* (BF, *N* = 12)

	LTL	BF	SED	*P*-value
Cooking loss, %	20.9	21.8	1.24	0.499
WBSF, N	26.3	31.4	1.54	0.004
Hardness, N	30.8	33.1	1.90	0.246
Cohesiveness	0.445	0.449	0.0082	0.613
Adhesiveness, Nmm	6.54	6.81	0.789	0.735
Springiness	0.737	0.765	0.0158	0.094
Chewiness, N	10.2	11.5	0.86	0.146
Resilience	0.431	0.453	0.0107	0.047
Collagen content, mg/g	4.95	6.68	0.419	<0.001
Insoluble collagen, mg/g	4.52	6.15	0.400	<0.001
Percentage of type III collagen, %	27.4	24.0	2.94	0.265
Proteoglycan content, mg/g collagen	18.3	17.1	3.29	0.720
IMF, %	1.10	1.16	0.198	0.762

**Figure 1.**
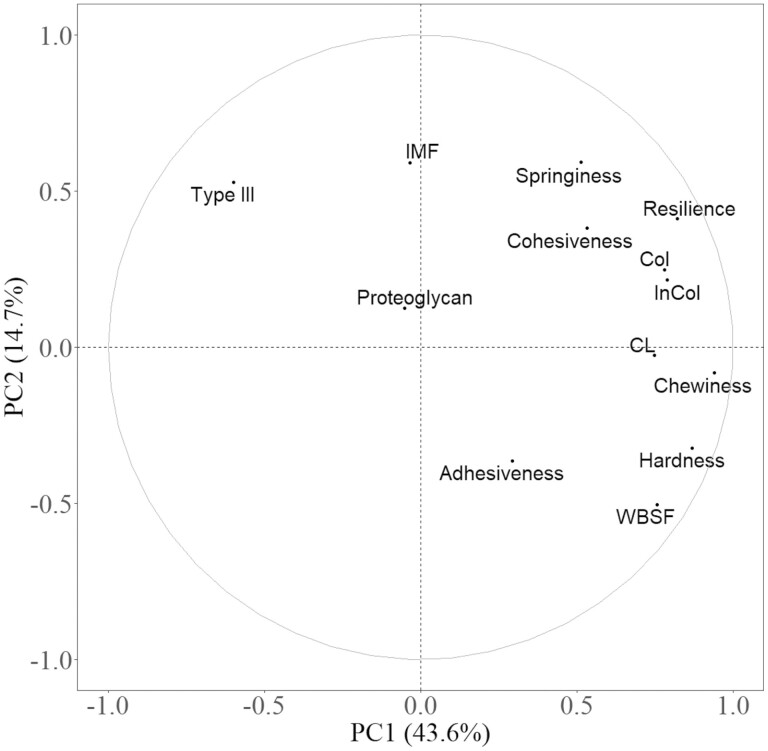
Principal component analysis (PCA) loading plot of different variables on both muscles. CL = cooking loss, WBSF = Warner–Bratzler shear force, Col = collagen content, InCol = insoluble collagen content, Type III = percentage of type III collagen, IMF = intramuscular fat.

The PCA plot of the variables for both muscles showed that principal component (PC) 1 explained 43.6% of variations and it was mainly characterized by chewiness, hardness, resilience, cooking loss, WBSF, cohesiveness, springiness, as well as total and insoluble collagen content. PC 2 explained 14.7% of variations and it was mainly determined by IMF (Figure 1). The correlation matrix exhibited that both total and insoluble collagen content were positively correlated (*P* < 0.05) with WBSF, hardness, cohesiveness, springiness, chewiness, and resilience (Table 2). The correlation coefficients of total and insoluble collagen content were similar. The highest correlation coefficients for both total and insoluble collagen were with resilience (*r* = 0.63 and 0.63, respectively), followed by chewiness (*r* = 0.58 and 0.59, respectively). The percentage of type III collagen was negatively correlated (*P* < 0.05 for all) with cooking loss (*r* = −0.42), WBSF (*r* = −0.68), hardness (*r* = −0.58) and chewiness (*r* = −0.52). No significant correlations were found between texture parameters and proteoglycan content or IMF.

**Table 2.** Correlation coefficients between IMF, collagen characteristics, and texture parameters of *Longissimus thoracis et lumborum* (LTL) and *Biceps femoris* (BF)

	Cooking loss, %	WBSF, N	Hardness, N	Cohesiveness	Adhesiveness, N mm	Springiness	Chewiness, N	Resilience
*Both muscles*								
Collagen content, mg/g	0.41	0.47*	0.49*	0.50*	0.10	0.36	0.58***	0.63**
Insoluble collagen, mg/g	0.42*	0.49*	0.50*	0.50*	0.11	0.35	0.59**	0.63**
Percentage of type III collagen (%)	−0.42*	−0.68***	−0.58**	−0.23	−0.21	0.08	−0.52*	−0.28
Proteoglycan ­content, mg/g ­collagen	0.22	−0.08	0.09	−0.30	−0.04	0.21	0.05	−0.04
IMF, %	−0.17	−0.22	−0.08	−0.02	0.08	0.38	0.02	0.04
*LTL*								
Collagen content, mg/g	0.41	0.75**	0.61*	0.17	0.18	0.04	0.59	0.33
Insoluble collagen, mg/g	0.52	0.81**	0.69*	0.22	0.16	0.07	0.67*	0.42
Percentage of type III collagen, %	−0.59	−0.72*	−0.68*	−0.61*	0.15	−0.13	−0.72*	−0.74**
Proteoglycan ­content, mg/g ­collagen	0.21	-0.08	0.03	-0.36	−0.30	0.25	0.01	0.06
IMF, %	−0.48	−0.44	−0.43	−0.35	−0.17	0.26	−0.41	−0.20
*BF*								
Collagen content, mg/g	0.46	0.09	0.40	0.74**	0.03	0.28	0.54	0.63*
Insoluble collagen, mg/g	0.40	0.09	0.39	0.72**	0.04	0.24	0.51	0.59*
Percentage of type III collagen, %	−0.10	−0.58*	−0.45	0.06	−0.37	0.34	−0.29	0.11
Proteoglycan ­content, mg/g ­collagen	0.31	−0.02	0.21	−0.26	0.10	0.27	0.15	−0.09
IMF, %	0.43	0.00	0.31	0.23	0.21	0.52	0.45	0.20

*P < 0.05, **P < 0.01, ***P < 0.001.

When considering individual muscles, total and insoluble collagen content as well as the percentage of type III collagen were correlated with different texture parameters in the two different muscles. Collagen content was positively correlated (*P* < 0.05) with WBSF (*r* = 0.75) and hardness (*r* = 0.61), while insoluble collagen content was positively correlated with WBSF (*r* = 0.81), hardness (*r* = 0.69) and chewiness (*r* = 0.67) in LTL (Table 2). Percentage of type III collagen was negatively correlated with WBSF (*r* = −0.72), hardness (*r* = −0.68), cohesiveness (*r* = −0.61), chewiness (*r* = −0.72) and resilience (*r* = −0.74) in LTL.

In BF, both total and insoluble collagen content were correlated (*P* < 0.05) with cohesiveness and resilience, and the highest correlation coefficient was with cohesiveness (*r* = 0.74 and 0.72, for total and insoluble collagen, respectively) (Table 2). The percentage of type III collagen was negatively correlated with WBSF (*r* = 0.58). No significant correlation was found between other parameters.

## DISCUSSION

The results confirm that pork BF had higher WBSF as well as total and insoluble collagen content than LTL. Total and insoluble collagen content and the percentage of type III collagen contributed to pork texture in both muscles, but the relationships differ between muscles. Therefore, the hypotheses were partially accepted.

Generally, the BF is tougher than LTL. The cooking loss, WBSF and TPA results of the two muscles in the present study were typical of pork LTL and BF (Choe et al., 2016; Hwang et al., 2003; Lachowicz et al., 2003). Total and insoluble collagen content of BF were higher than LTL, which also agreed with the literature (Lebret et al., 2015; Nishimura et al., 2009; Wheeler et al., 2000). This difference is attributed to their functions as LTL is a positional muscle and BF is a locomotive muscle (Dubost et al., 2013). During physical activity, the functional effort of locomotive muscle is greater than positional muscle because connective tissues facilitate force transmission (Purslow, 2010). Therefore, BF has higher total and insoluble collagen content than LTL. Few studies investigated the percentage of type III collagen and proteoglycan content of pork muscles. Types I and III collagen are the predominant isoforms in skeletal muscle and their relative amount is proposed to contribute to the mechanical strength of meat (Bailey et al., 1979; Stanton and Light, 1987). Proteoglycans forms a network to interact with collagen fibrils, which stabilizes the structure of connective tissue. The degradation of proteoglycan during post-mortem aging is related to the increase in meat tenderness (Nishimura et al., 1996). It is noted that pork samples in this study were not aged, and the post-slaughter time was unknown. To the best of our knowledge, there is no previous study on pork proteoglycans. The present findings on proteoglycans fell within the range for beef LTL and BF (Dubost et al., 2013; Mikołajczak et al., 2019; Stanton and Light, 1990), but they did not differ between pork LTL and BF in this study. In the present study, IMF content did not differ between muscles. The IMF content of pork LTL and BF did not differ in some previous studies (Leseigneur-Meynier and Gandemer, 1991; Martins et al., 2012), while another study reported that pork BF had higher IMF content than LTL (Kim et al., 2008). The conflicting report on IMF content between LTL and BF is likely due to differences in genotype, sex, raising condition, and body weight of the pigs in these studies (Font-i-Furnols et al., 2019). In conclusion, pork LTL and BF differed in texture and collagen characteristics but not in IMF.

Regardless of muscles, pork texture is affected by total collagen content and its solubility. Correlation results showed that total and insoluble collagen content were mostly correlated with resilience, followed by chewiness. Resilience reflects the ability of the sample to regain its original height and chewiness is the energy required to chew the sample (Wee et al., 2018). Collagen has rubber-link properties (Lepetit, 2007). Upon cooking to above 60 °C, collagen contracts and shows high elasticity. The contraction of collagen decreases the elastic modulus of meat, while it increases the resistance of muscle fibers. The balance between these two effects determines the elastic modulus of collagen which contributes to the toughness of meat (Lepetit, 2007). As a result, pork with higher collagen content is more elastic and requires more energy to chew. Resilience and chewiness were mostly related to collagen content. Total and insoluble collagen content were also moderately correlated with WBSF, hardness, and cohesiveness, indicating that collagen only partly contributed to cooked meat toughness.

Most of the texture parameters were better predicted by insoluble collagen content, although the correlation coefficients for total and insoluble collagen content were similar in both and individual muscles. Purslow (2014) proposed that there were two proportions of collagen in which one portion was easily degraded by aging and cooking and it did not contribute to the strength of connective tissue and thus, meat toughness. The other fraction was more cross-linked and heat insoluble, which determined the strength of cooked meat (Purslow, 2014). The findings of the present study agree with this hypothesis. Therefore, the contribution of collagen to cooked pork texture depends on the insoluble portion. The properties of the insoluble portion need further investigation.

The effects of different types of collagen on pork texture remained unclear. In this study, the percentage of type III collagen was negatively correlated with WBSF, hardness, and chewiness. Previous studies on beef showed conflicting results on the relationship between the proportion of type III collagen and meat tenderness. Burson and Hunt (1986a) and Maiorano et al. (2000) found that muscles with a higher percentage of type III collagen were more tender, while Bailey et al. (1979) reported the opposite. McCormick (1999) suggested that type III collagen was the embryonic form of collagen and its concentration was higher in newly synthesized and less cross-linked collagen. As animals grow, type III collagen is converted to type I collagen (McCormick, 1999). On the other hand, Burson and Hunt (1986b) pointed out that type I collagen was more heat soluble than type III collagen. The mechanisms of how collagen isoforms affect pork tenderness need further investigation, although the percentage of type III collagen was negatively correlated with WBSF in the present study.

The exact role of proteoglycans in meat tenderness is unknown. The present study failed to show a significant correlation between texture parameters and proteoglycan content, so hypothesis 2 was partly rejected. Wang et al. (2016) reported that proteoglycan content was positively correlated with WBSF in beef, while Listrat et al. (2020) found that proteoglycan content was positively correlated with sensory tenderness only in M. semitendinosus. The insignificant correlation of proteoglycan found in the present study is probably explained by their low concentration and low elastic modulus (Lepetit, 2007; Listrat et al., 2020). Proteoglycan has little influence on pork texture in this study.

IMF might contribute to pork tenderness, but it was not shown by the present study. Several studies demonstrated the contribution of IMF to pork instrumental and sensory tenderness, although the correlations were weak or breed-dependent (Blanchard et al., 2000; van Laack et al., 2001; Wood et al., 2004). Nishimura et al. (1999) observed that IMF tenderized meat by disrupting the perimysial connective tissue during development, leading to the weakening of the perimysial structure and the dilution of collagen fibers. However, the authors also mentioned that this effect was only observed in meat with IMF higher than 8% (Nishimura et al., 1999). In the present study, the IMF content of pork was low within a small range. More samples with a greater range in IMF content are needed to show the effect of IMF on pork tenderness.

The contribution of collagen characteristics to pork texture differed between muscles. However, total and insoluble collagen content was correlated with some texture parameters in both LTL and BF, which was different from our hypothesis. Wheeler et al. (2000) reported that collagen content was negatively correlated with sensory tenderness in five pork muscles but was not correlated with tenderness within individual muscles, including LTL and BF. Wheeler et al. (2002) also found no significant correlation between collagen content and WBSF in pork LTL. Instrumental measurement of meat tenderness did not truly reflect the sensory tenderness (Warner et al., 2021). The small differences in texture shown by an instrument may not be detected by consumers. Therefore, sensory evaluation may be required to further define the contribution of collagen to pork texture in different muscles.

## CONCLUSIONS

The BF was tougher than LTL, partly because of higher total and insoluble collagen content. Total and insoluble collagen content positively contributed to the toughness of cooked pork and the percentage of type III collagen was positively correlated with pork tenderness in both muscles as well as in the LTL or BF itself. Neither proteoglycans nor IMF content were correlated with pork texture parameters, possibly due to their low concentration. However, instrumental tenderness may not be a good predictor of connective tissue contribution to cooked meat toughness. Future studies should be conducted to investigate the relationship between collagen characteristics and sensory tenderness with more carcass replication and different muscles.

## Supplementary Material

txac129_suppl_Supplementary_Material

## Abbreviations

BF: biceps femoris
IMF: intramuscular fat
LTL: Longissimus thoracis et lumborum
PC: principal component
PCA: principal component analysis
TPA: texture profile analysis
WBSF: Warner-Bratzler shear force
