# Supplementary material for: Collagen characteristics affect the texture of pork Longissimus and Biceps femoris
Source: Transl Anim Sci. 2022 Sep 20;6(4):txac129. doi: 10.1093/tas/txac129 (PMC9558871; doi:10.1093/tas/txac129)
Supplement: txac129_suppl_Supplementary_Material [file txac129_suppl_supplementary_material.docx]

LTL3

LTL2

LTL1

75kDa

50kDa

BF1

BF2

BF3

α2(I)

α1(I)

α1(III)

Ladder

Type III standard

Figure S1. Original image of SDS-PAGE on *Longissimus thoracis et lumborum* (LTL) and *Biceps femoris* (BF). LTL1-3 are LTL samples from three different animals. BF1-3 are BF samples from three different animals. α1(III) is the α1 chain of type III collagen. α1(I) is the α1 chain of type I collagen. α2(I) is the α2 chain of type I collagen.

150kDa

250kDa

100kDa


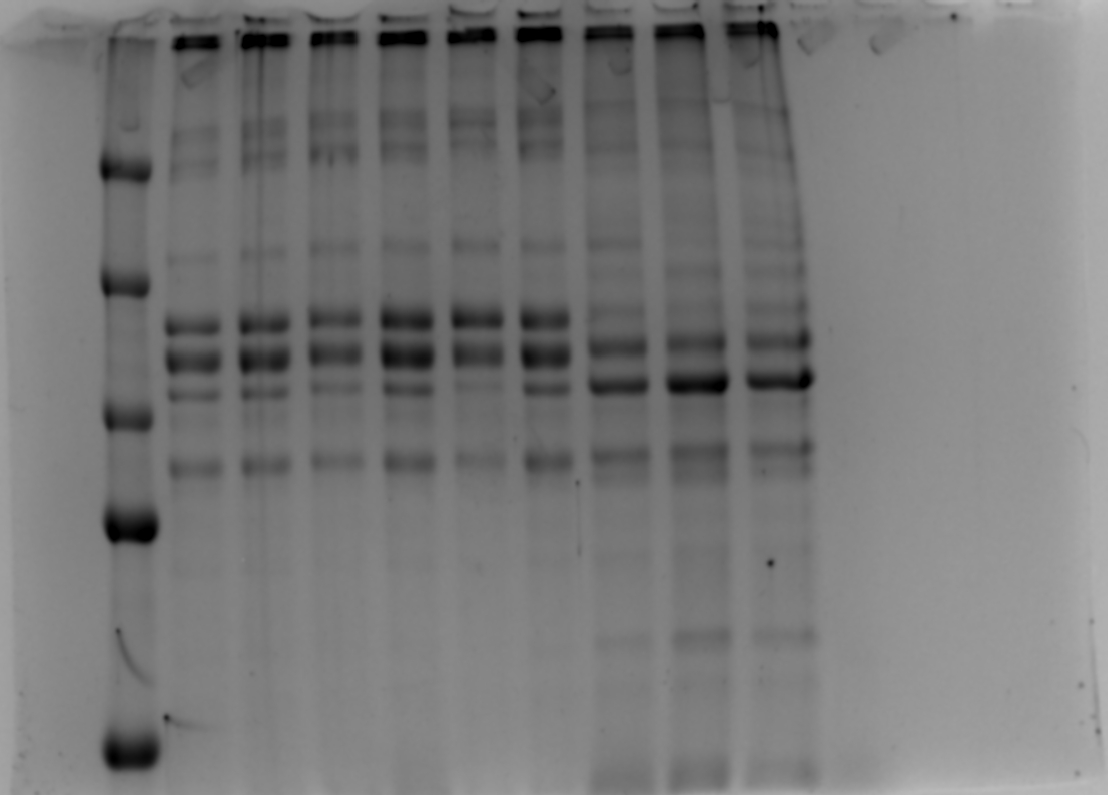


Ladder

BF4

BF5

BF6

BF7

BF8

BF11

BF9

BF10

BF12

75kDa

50kDa


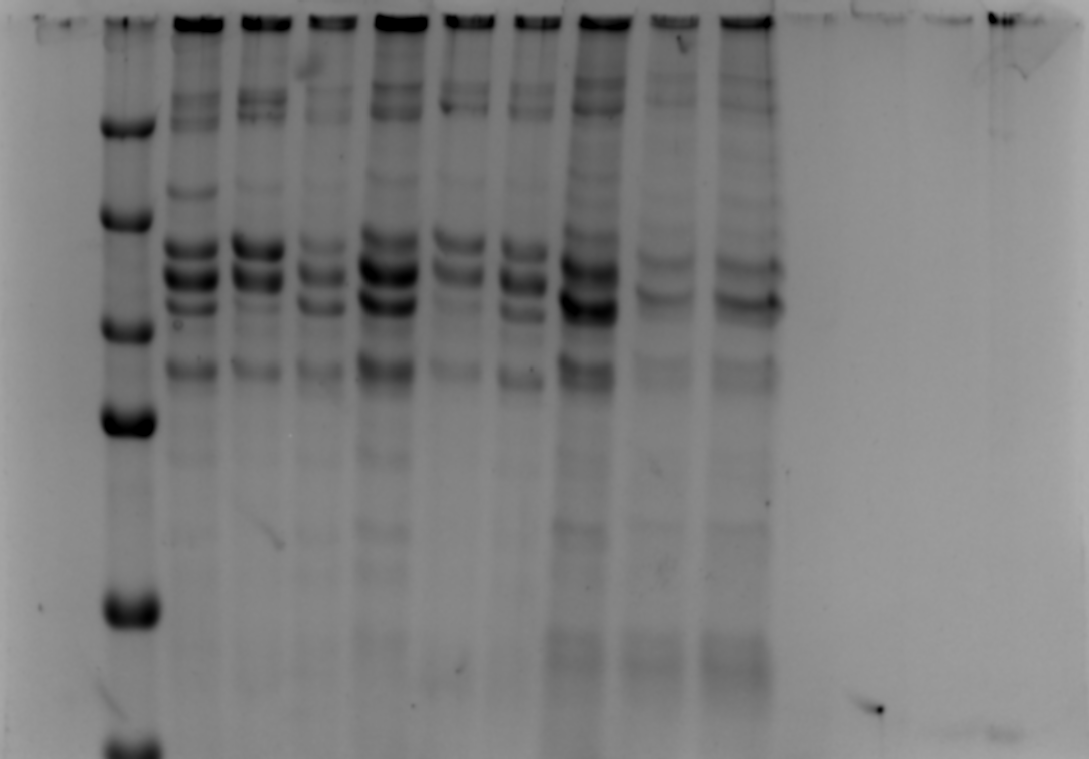


α1(I)

α1(III)

α2(I)

100kDa

150kDa

250kDa

Figure S2. Original image of SDS-PAGE on *Biceps femoris* (BF). BF4-12 are BF samples from three different animals. α1(III) is the α1 chain of type III collagen. α1(I) is the α1 chain of type I collagen. α2(I) is the α2 chain of type I collagen.

α2(I)

α1(III)

α1(I)

LTL8

LTL9

LTL6

LTL10

LTL5

LTL11

LTL12

Figure S3. Original image of SDS-PAGE on *Longissimus thoracis et lumborum* (LTL). LTL4-12 are LTL samples from three different animals. LTL11 was omitted from analysis. α1(III) is the α1 chain of type III collagen. α1(I) is the α1 chain of type I collagen. α2(I) is the α2 chain of type I collagen.

Ladder

LTL4

LTL7

250kDa

150kDa

100kDa

75kDa

50kDa
